# Aggregation of type-2 diabetes, prediabetes, and metabolic syndrome in German couples

**DOI:** 10.1038/s41598-024-53417-1

**Published:** 2024-02-05

**Authors:** Lara Brieger, Sara Schramm, Börge Schmidt, Ulla Roggenbuck, Raimund Erbel, Andreas Stang, Bernd Kowall

**Affiliations:** 1 Institute for Medical Informatics, Biometry and Epidemiology, Medical Faculty, University Duisburg-Essen, Essen, Germany; 2 School of Public Health, Department of Epidemiology Boston University, 715 Albany Street, Talbot Building, Boston, MA 02118 USA

**Keywords:** Diseases, Health care, Risk factors

## Abstract

We aimed to examine the concordance of type-2 diabetes, prediabetes and the metabolic syndrome in couples. In cross-sectional analyses, we used data from 1173 couples with index persons from the Heinz Nixdorf Recall Study (2011–2015), a population-based cohort study in Western Germany, and partners from the associated Heinz Nixdorf Multigeneration Study (2013–2016). Mean age (standard deviation) was 67.2 (6.6) years in index persons, and 67.8 (7.7) years in partners. The exposure was the presence of diabetes, prediabetes or metabolic syndrome in index persons, the outcome was the presence of the same health status in partners. Diabetes was defined by either self-reported diagnosis, intake of antidiabetic drugs or insulin, or HbA1c ≥ 6.5%. If the index person had prediabetes or diabetes, the partner was 1.46 (95% CI 1.07–2.00) times more likely to have diabetes than partners of index persons without the condition in the crude model (adjusted model: 1.33 (0.97–1.83)). For self-reported diabetes and for the metabolic syndrome, the corresponding prevalence ratios were 1.33 (0.90–1.97) and 1.17 (1.03–1.32), respectively (adjusted models: 1.23 (0.77–1.94), 1.04 (0.91–1.18)). In German couples, there was weak to moderate concordance of type-2 diabetes, prediabetes and the metabolic syndrome in crude, but poor concordance in adjusted models.

## Introduction

In 2021, 537 million persons worldwide had diabetes, and this figure is expected to increase by 46% by 2045^1^. People with diabetes have a two-fold risk of vascular diseases and a higher risk of premature death than persons without diabetes^2,3^. With regard to the adverse consequences of diabetes, preventing or delaying diabetes is of great importance.

Spousal association for diabetes or prediabetes might bring partners of persons with diabetes to have their glycaemic status checked and to engage in changes of lifestyle. Concordance of diabetes has been reported in several studies^4–8^. In a recent meta-analysis, spouses of persons with type-2 diabetes were shown to have higher odds of type-2 diabetes (pooled odds ratio = 1.88 (95% confidence interval (CI) 1.52–2.33)^6^. However, results are rather inconsistent ranging from small effects in a study from China (OR = 1.1 (1.0–1.3)) to huge effects reported in a study from the USA (OR = 8.7 (7.4–10.2))^9,10^.

Identifying people with unknown prediabetes is important because people with prediabetes are at increased risk of diabetes, but also have the opportunity to reduce this risk of progression to diabetes through lifestyle interventions^11,12^. The metabolic syndrome is also of interest, as it includes various risk factors (high blood pressure, increased blood lipid levels and waist circumference, dysgylcaemia) for cardiovascular events such as heart attacks and strokes^13^.

Little is known about spousal concordance for prediabetes and for the metabolic syndrome. Apart from few studies from Asia and the UK, the association of prediabetes in married couples has not been paid much attention^14–16^. Studies from Korea and Japan found spousal concordance for the metabolic syndrome^14,17^.

Even though there are studies suggesting genetic similarities between partners^18^, a study of over 47,000 pairs from the UK Biobank indicates that estimates of genetic associations are smaller for spouses than estimates for random pairs for height, education, and BMI^19^. Therefore, we assume that similarities in dysglycemia status in couples can be attributed mostly to a shared environment, a shared lifestyle, similar health care seeking behaviour, or to criteria in the choice of partner ("assortative mating")^20^.

In the present study, we aim to investigate spousal concordance of prevalent type-2 diabetes, prediabetes, and the metabolic syndrome in a large German population of 1173 couples. Furthermore, we aim to study spousal concordance of risk factors like obesity, waist circumference, lipids, smoking and physical inactivity.

## Methods

### Study population

The analyses were based on data from the Heinz Nixdorf Recall (HNR) Study and the Heinz Nixdorf Multigeneration Study (MGS). The HNR study is a large ongoing prospective population-based cohort study with 4814 participants conducted in three cities in the Ruhr area (Mülheim, Essen, Bochum) in Germany starting in 2000. The study objective and design are described in more detail elsewhere^21^. Participants who were almost completely Caucasians aged 45 to 74 years were randomly selected using population registration data (recruitment efficacy proportion: 55.8%)^22^. The first visit to the study center took place between 2000 and 2003 (T0). Participants were re-examined after 5 (2005–2008 (T1)) and 10 years (2011–2015 (T2)).

In addition, 1237 partners and 1660 children of the participants of the HNR Study were examined in the years 2013 to 2016 in the MGS (T0). In the present cross-sectional study, data from the HNR Study at T2 and data from the MGS (T0) were examined.

Data for both studies were collected using questionnaires and computer-assisted personal interviews (CAPI). In addition, a physical examination was performed which included anthropometric measurements and a blood sample. In the following we refer to index persons and their partners: Index persons are participants in the HNR Study and partners are participants in the MGS. Both studies were approved by the local Ethics Committee of the University of Essen. Written informed consent had to be obtained beforehand^21,23^. All methods were performed in accordance with the relevant guidelines and regulations.

**Figure 1:**
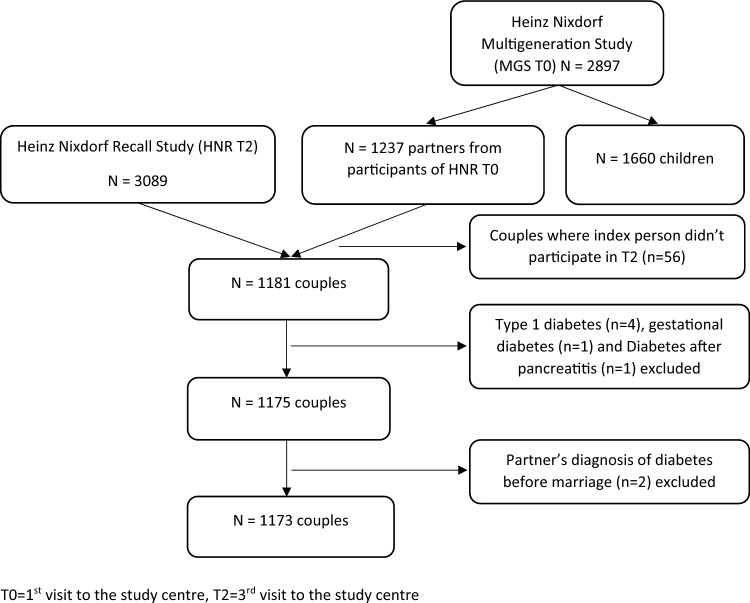
Flowchart of selection of the study sample.

A total of 1173 couples, including three homosexual couples, were included in the current study (Fig. 1). Persons with type-1 diabetes were excluded. Type-1 diabetes was defined by a self-report of type-1 diabetes or by age at diabetes onset below 30 years. Participants from the MGS were invited as partners of the index persons at MGS baseline (T0). Index persons from the HNR and their partners from MGS were viewed as couples, even if in 80 (6.8%) cases information on marital status of the index person and the partner differed. These discordances can be explained by the fact that index persons and their partners were not interviewed exactly at the same time, and that death or separation may have occurred in the meantime. Nevertheless, these discordant cases were treated as couples in the study.

### Definition of the study variables

Prevalent type-2 diabetes was defined by a self-report of diabetes, intake of antidiabetic drugs (ATC A10) or insulin, or HbA1c ≥ 6.5%^24^. Prediabetes was defined by 5.7% ≤ HbA1c < 6.5%^24^.

To define the metabolic syndrome, the criteria of the "Joint Interim Statement" were used^25^. However, due to lack of data on all criteria, some adjustments were made. If the fasting status of the participant was not available or the participant was not fasting, a triglyceride value of ≥ 175 mg/dl was used instead of ≥ 150 mg/dl^26^. Guideline values for waist circumference for European and US populations, respectively, were used (≥ 88 cm for women and ≥ 102 cm for men). Instead of the criterion of fasting glucose ≥ 100 mg/dl, we used HbA1c ≥ 5.7% or intake of diabetes medication or self-report of diabetes.

BMI was determined from the body weight (kg) divided by the square of the height (m^2^) of the participants. Data on weight were collected using scales from the company “Seca” (seca GmbH & Co. kg, Hamburg, Germany). Waist circumference was measured at the level of the narrowest point between the lowest rib and the iliac crest to the nearest 0.1 cm using a flexible, non-elastic measuring tape. The participants wore light underwear^27^. To determine blood pressure, three blood pressure measurements were taken in each case using an automatic oscillometric measuring device (Omron HEM-705CP; OMRON Corporation, Hoofdorp, Netherlands). The mean value from the 2nd and 3rd measurement was used for the study^28^. The analyses of the blood samples were carried out in the central laboratory of the University Hospital Essen. Blood samples were immediately stored at 4 degrees Celcius after collection and analyzed within 12 h at the central laboratory of the University Hospital of Essen, Germany. Various parameters were measured, including cholesterol, HDL, LDL, and triglycerides using the laboratory device “ADVIA Clinical Chemistry” by Siemens. The HbA1c value was measured using a nephelometer (BN-II, Dade-Behring Inc.; Deerfield, IL, USA)^29^.

Information on marital status, education, alcohol consumption, diet, smoking status, sports, health status and medication use were collected through interviews and questionnaires. Education was categorised as no degree, main or elementary school, secondary or polytechnical school, advanced technical college entrance and higher educational entrance qualification^27^. Average alcohol consumption (g/day) was estimated based on glasses of beer, wine, sparkling wine, and liquor drunk per week. The following calculation was used: amount of beverage (ml) x (volume percentage of beverage/100) x specific weight of alcohol (0,794 g/cm^3^). Based on the Food Frequency Questionnaire, a validated dietary pattern index was derived, which follows the guidelines of the German Society of Nutrition for a cardio-protective diet^30,31^. This index ranges from 0 to 26 points. The higher the score, the more likely the diet meets the guidelines. Smokers were divided into three categories (current, former, never). Smokers were divided into three categories (current, former, never). For the survey of sports, participants were asked what kind of sports they had practised in the last four weeks. Participants were considered as practicing sports if they stated that they had practiced any kind of sport at least once in the last four weeks. Subjective health status was categorised as very good, good, satisfactory, not good, or bad. Participants were asked to bring the medication they had taken in the last 7 days before the examination^29^.

### Statistical analyses

Characteristics of index persons and their partners were compared using means (standard deviations), medians (first quartile, third quartile), and proportions. We estimated prevalence ratios instead of odds ratios because the rare disease assumption was not fulfilled for many outcomes (i.e. the outcome was present in more than 10% of the persons in one category). If the rare disease assumption is not fulfilled this can lead to odds ratios which are much larger than the corresponding prevalence ratios. Log-binomial regression models were fitted to estimate prevalence ratios (PRs) with 95% confidence intervals for two kinds of associations: (1) associations between risk factors for type-2 diabetes in the index person as exposure and the corresponding risk factors in the partner as outcome, (2) associations between disease status of the index person (type-2 diabetes, prediabetes, metabolic syndrome) as exposure and the corresponding disease status of the partner as outcome. In addition to crude analyses (model 1), three adjusted models were fitted: Model 2 was adjusted for age and education, model 3 for smoking, sports, diet and alcohol consumption, and model 4 for BMI and blood lipids (cholesterol, HDL, LDL, triglycerides). In a first sensitivity analysis, only married couples were included. In a second sensitivity analysis, separate analyses were done only with men and only with women, respectively, as index persons. We did complete case analyses. If log-binomial models did not converge, log-linear models with a Poisson working likelihood and robust standard errors were used.

In Germany, around 20% of adults with diabetes are not aware of it^1,32^. Therefore, self-reported diabetes may be subject to misclassification, which we accounted for in a quantitative bias analysis by using a precast Excel Misclassification Spreadsheet^33–35^. We assumed that the sensitivity of self-reported diabetes in index and partners varied between 0.80 and 0.90, while the specificity of self-reported diabetes was close to 1 (0.98 or 1), because people without diabetes very rarely report having it. The quantitative bias analysis shows what the true prevalence ratios would be if the assumptions about sensitivity and specificity were correct. In a multidimensional analysis, we considered misclassification bias both of the exposure (i.e. type-2 diabetes in the index person), and of the outcome (i.e. type-2 diabetes in the partner). We considered differential and non-differential misclassification – differential misclassification means that, e.g. the sensitivity of type-2 diabetes in the index person depends on whether the partner has type-2 diabetes or not.

The analyses were calculated using the statistical programme SAS 9.4 (SAS Institute Inc., Cary, NC, USA).

## Results

The 1173 index persons and 1173 partners were on average 67.2 ± 6.6 and 67.8 ± 7.7 years old, respectively (Table 1). 1080 (92.1%) couples were married and lived together. On average, married couples were married for 39.7 years. 14.4% of the index persons and 12.1% of the partners reported having diabetes. When the HbA1c value and diabetes medication were additionally used as criteria of type-2 diabetes, 17.6% of the index persons and 14.5% of the partners were classified as having type-2 diabetes. This suggests that slightly less than 20% of persons with type-2 diabetes were unaware of having the disease (e.g. (17.6–14.4)/17.6 = 0.182 in the index persons). According to the Joint Interim Statement definition^25^, 54.3% of the index persons and 48.1% of the partners had the metabolic syndrome.

**Table 1:** Characteristics of index persons and their partners: the Heinz Nixdorf recall (HNR) study and the Heinz Nixdorf Multigeneration study (MGS).

		Index persons (HNR T2)	Partners (MGS T0)
N		1173	1173
Age (years)		67.2 ± 6.6	67.8 ± 7.7
	Median (q1, q3)	67 (62, 72)	68 (62, 73)
	Range	55–85	27–90
Sex (male) (%)^a^		668 (57.0%)	508 (43.3%)
Marital status (%)	Married (living together)	1080 (92.1%)	
	Not married (living together)	13 (1.1%)	
	Inconsistent information	80 (6.8%)	
Duration of marriage (years)		39.7 ± 13.1	
	Median	43	
	< 5	18 (1.5%)	
	≥ 5 < 10	19 (1.6%)	
	≥ 10 < 20	83 (7.1%)	
	≥ 20 < 40	290 (24.7%)	
	≥ 40 < 60	670 (57.1%)	
BMI (kg/m^2^)		28.0 ± 4.8	27.9 ± 4.6
Waist circumference (cm)		96.5 ± 13.4	96.3 ± 13.4
Cholesterol (mg/dl)		214.1 ± 40.6	214.6 ± 41.6
HDL (mg/dl)		61.1 ± 17.4	62.9 ± 18.1
LDL (mg/dl)		128.7 ± 34.7	130.7 ± 35.4
Triglycerides (mg/dl)		130.5 ± 78.4	127.2 ± 82.4
	Median (q1, q3)	111 (82,159)	107 (78,152)
HbA1c [%]		5.9 ± 0.7	5.8 ± 0.6
Systolic BP (mmHg)		127.4 ± 18.1	129.5 ± 16.9
Diastolic BP (mmHg)		75.4 ± 9.7	73.1 ± 10
Intake of cholesterol-lowering drug	Yes	340 (29.0%)	314 (26.8%)
	No	833 (71.0%)	859 (73.2%)
Intake of antihypertensive drug	Yes	631 (53.8%)	596 (50.8%)
	No	542 (46.2%)	577 (49.2%)
Intake of antidiabetics	Yes	127 (10.8%)	97 (8.3%)
	No	1046 (89.2%)	1076 (91.7%)
Intake of insulin	Yes	35 (3.0%)	22 (1.9%)
	No	1138 (97.0%)	1151 (98.1%)
Dietary pattern index (0–26)^b^		12.8 ± 3.1	12.6 ± 3
Sports^c^	Yes	767 (65.4%)	492 (41.9%)
	No	405 (34.5%)	399 (34.0%)
	Missing	1 (0.1%)	282 (24.0%)
Alcohol (g/day)		14.2 ± 21.0	9.3 ± 14.0
	Median	6.6	3.9
Smoking	Never	482 (41.1%)	523 (44.6%)
	Former	557 (47.5%)	491 (41.9%)
	Current	132 (11.3%)	153 (13.0%)
General health status in the last 12 months	Very good	47 (4.0%)	87 (7.4%)
	Good	495 (42.2%)	442 (37.7%)
	Satisfactory	458 (39.0%)	446 (38.0%)
	Not good	144 (12.3%)	153 (13.0%)
	Bad	28 (2.4%)	39 (3.3%)
Education	None	11 (0.9%)	90 (7.7%)
	Main or elementary school	572 (48.8%)	478 (40.8%)
	Secondary or polytechnic school	234 (19.9%)	284 (24.2%)
	Advanced technical college entrance	92 (7.8%)	73 (6.2%)
	Higher educational entrance qualification	263 (22.4%)	240 (20.5%)
Diabetes (self-report)	Yes	169 (14.4%)	142 (12.1%)
	No	1004 (85.6%)	1031 (87.9%)
Diabetes (by self-report, drug intake or HbA1c of ≥ 6.5%)	Diabetes	207 (17.6%)	170 (14.5%)
	Prediabetes^d^	531 (45.3%)	455 (38.8%)
	No	418 (35.6%)	510 (43.5%)
Metabolic syndrome^e^	Yes	637 (54.3%)	564 (48.1%)
	No	509 (43.4%)	559 (47.7%)

Mean ± standard deviation; median (first quartile, third quartile); n (proportions (%)).

*HNR T2* Heinz Nixdorf Recall Study 3rd visit to the study centre (2011–2014), *MGS* MultiGeneration Study (2013–2016), *BP* blood pressure.

^a^The sum of male index persons and male partners is 1176 because there are 3 homosexual couples.

^b^The higher the score, the more likely the diet meets the guidelines,

^c^Participants were considered as practicing sports if they stated that they had practiced any kind of sport at least once in the last four weeks,

^d^Prediabetes is defined by 5.7% ≤ HbA1c < 6.5%,

^e^The metabolic syndrome is defined by a modification of the Joint Interim Statement^25^.

Partners of index persons with obesity had a higher prevalence of being obese than partners of index persons without obesity (PR = 1.52 [95% CI 1.26–1.83]) (Table 2). Similar results were observed for increased waist circumference (≥ 88 for women/ ≥ 102 for men) (PR = 1.27 [95% CI 1.14–1.42]) and decreased HDL levels (< 40 for men/ < 50 for women) (PR = 1.18 [95% CI 0.75–1.86]). If the index person took lipid-lowering drugs, antihypertensive drugs, antidiabetic drugs, or insulin, the prevalence of the partner to take the same drugs was increased. This is the case for cholesterol-lowering drugs (PR = 1.56 [95% CI 1.29–1.88]) and antihypertensive drugs (PR = 1.30 [95% CI 1.16–1.46]). For components of lifestyle strong spousal concordance is observed: index persons practicing sports were more likely to have partners practicing sports also (PR = 1.61 [95% CI 1.39–1.87]), and partners were more likely to be current smokers if they were married to a current smoker (PR = 4.17 [95% CI 2.87–6.05]). Furthermore, strong spousal correlation was observed for low education (PR = 2.34 [95% CI 2.04–2.68]).

**Table 2:** Unadjusted prevalence ratios (95% confidence interval) for spousal associations of risk factors for (pre-)diabetes/metabolic syndrome: Results from log-binomial regression analysis.

		Index person exposure (n)	Number of partners with risk factor or intake of same drug (n) (%)	Prevalence ratio (95% CI)
Obesity (kg/m^2^)	≥ 30	313	116 (37.1%)	1.52 (1.26–1.83)^a^
	< 30	848 (ref)	207 (24.4%)	1
Waist circumference (cm)	≥ 88 (f)/102 (m)	579	338 (58.4%)	1.27 (1.14–1.42)
	< 88 (f)/102 (m)	585 (ref)	269 (46.0%)	1
HDL (mg/dl)	< 40 (m)/50 (f)	122	18 (14.8%)	1.18 (0.75–1.86)
	≥ 40 (m)/50 (f)	1005 (ref)	126 (12.5%)	1
Triglycerides (mg/dl)^b^	≥ 150	290	73 (25.2%)	1.04 (0.82–1.31)
	< 150	833 (ref)	202 (24.3%)	1
Intake of cholesterol-lowering drug	Yes	340	122 (35.9%)	1.56 (1.29–1.88)
	No	833 (ref)	192 (23.0%)	1
Intake of antihypertensive drug	Yes	631	359 (56.9%)	1.30 (1.16–1.46)
	No	542 (ref)	237 (43.7%)	1
Intake of antidiabetics	Yes	127	14 (11.0%)	1.39 (0.81–2.37)
	No	1046 (ref)	83 (7.9%)	1
Intake of insulin	Yes	35	1 (2.9%)	1.55 (0.21–11.19)
	No	1138 (ref)	21 (1.8%)	1
Dietary pattern index^c^	< 13	554	328 (59.2%)	1.39 (1.24–1.56)
	≥ 13	585 (ref)	249 (42.6%)	1
Sports^d^	Yes	570	365 (64.0%)	1.61 (1.39–1.87)
	No	320 (ref)	127 (39.7%)	1
Smoking	Current	132	47 (35.6%)	4.17 (2.87–6.05)
	Former	553	65 (11.8%)	1.38 (0.95–1.99)
	Never	480 (ref)	41 (8.5%)	1
General health status in the last 12 months	Satisfactory/not good/bad	626	363 (58.0%)	1.14 (1.03–1.27)
	Very good/good	540 (ref)	274 (50.7%)	1
Education^e^	Low	577	395 (68.5%)	2.34 (2.04–2.68)
	Medium/high	587 (ref)	172 (29.3%)	1

n (proportions (%)), ref: reference group, *f* female, *m* male.

^a^**How to read**: Partners of index persons with obesity have a 1.52-fold higher prevalence of obesity than partners of index persons without obesity.

^b^High triglycerides are defined by either ≥ 150 mg/dl when sober or ≥ 175 mg/dl when not sober or not clear if sober.

^c^The higher the score, the more likely the diet meets the guidelines,

^d^Participants were considered as practicing sports if they stated that they had practiced any kind of sport at least once in the last four weeks.

^e^Low education = none/main or elementary school, medium/high education = secondary or polytechnic school/advanced technical college entrance/higher educational entrance qualification.

If the index person reported having diabetes, 15.4% of the partners also gave a self-report of diabetes whereas only 11.6% of partners of index persons without self-reported diabetes reported having diabetes (PR = 1.33 [95% CI 0.90–1.97]) (Table 3). If type-2 diabetes is defined by self-report, HbA1c or intake of antidiabetic drugs, 17% of partners of index persons with type-2 diabetes also had type-2 diabetes, but only 14.5% if the index person did not have type-2 diabetes (PR = 1.17 [95% CI 0.83–1.66). If the index person had prediabetes or type-2 diabetes, the prevalence of type-2 diabetes in the partners was increased by 46% (PR = 1.46 [95% CI 1.07–2.00]). If index persons had the metabolic syndrome, the prevalence was increased in their partners compared to partners of index persons without the metabolic syndrome (PR 1.17 [95% CI 1.03–1.32]). In the models 2 and 3 with adjustment for sociodemographic and lifestyle factors, effect estimates decreased for all associations. However, in the fourth model with additional adjustment for BMI and lipids, effect estimates increased for some associations in comparison with model 3 (Table 3).

**Table 3:** Prevalence ratios (95% confidence interval) for spousal association of (pre-)diabetes/metabolic syndrome: Results from log-binomial regression analyses.

Exposure index person	Outcome partner	Number of index persons with exposure in column 1	Number of partners with outcome in column 2	Model 1	Model 2	Model 3	Model 4
Diabetes (self-report)	Diabetes (self-report)	Yes 169	26 (15.4%)	1.33 (0.90–1.97)^f^	1.19 (0.81–1.76)	1.15 (0.78–1.71)	1.23 (0.77–1.94)^e^
		No 1004 (ref)	116 (11.6%)	1	1	1	1
Diabetes (self-report, drug intake, HbA1c)^a^	Diabetes (self-report, drug intake, HbA1c)^a^	Yes 200	34 (17.0%)	1.17 (0.83–1.66)	1.05 (0.75–1.48)	1.01 (0.72–1.42)	1.07 (0.74–1.54)
		No 918 (ref)	133 (14.5%)	1	1	1	1
Prediabetes^b^	(Pre-)Diabetes^c^	Yes 511	288 (56.4%)	1.14 (1.00–1.29)	1.08 (0.96–1.22)	1.07 (0.94–1.21)	1.08 (0.95–1.23)
		No 407 (ref)	202 (49.6%)	1	1	1	1
Diabetes (self-report, drug intake, HbA1c)^a^	(Pre-)Diabetes^c^	Yes 200	128 (64.0%)	1.20 (1.06–1.35)	1.16 (1.03–1.30)	1.13 (1.00–1.27)	1.12 (0.99–1.28)
		No 918 (ref)	490 (53.4%)	1	1	1	1
(Pre-)Diabetes^c^	(Pre-)Diabetes^c^	Yes 711	416 (58.5%)	1.18 (1.05–1.32)	1.12 (1.00–1.25)	1.10 (0.98–1.24)	1.10 (0.98–1.23)
		No 407 (ref)	202 (49.6%)	1	1	1	1
(Pre-)Diabetes^c^	Diabetes (self-report, drug intake, HbA1c)^a^	Yes 711	120 (16.9%)	1.46 (1.07–2.00)	1.28 (0.93–1.76)	1.26 (0.92–1.74)	1.33 (0.97–1.83)
		No 407 (ref)	47 (11.5%)	1	1	1	1
Metabolic syndrome^d^	Metabolic syndrome^d^	Yes 608	326 (53.6%)	1.17 (1.03–1.32)	1.07 (0.95–1.21)	1.06 (0.94–1.20)	1.04 (0.91–1.18)
		No 488 (ref)	224 (45.9%)	1	1	1	1

Model 1: without adjustment, Model 2: adjusted for age and education, Model 3: + adjustment for alcohol, dietary pattern index, smoking, sports, Model 4: + adjustment for BMI, lipids (HDL, LDL, cholesterol, triglycerides). n (proportions (%)).

*ref* reference group, *MS* metabolic syndrome.

^a^Diabetes is defined by either self-report, intake of antidiabetics or insulin or HbA1c ≥ 6.5%

^b^Prediabetes is defined by 5.7% ≤ HbA1c < 6.5%

^c^Prediabetes or diabetes.

^d^The metabolic syndrome is defined by a modification of the Joint Interim Statement^25^.

^e^Log-linear models with a Poisson working likelihood and robust standard errors were used.

^f^**How to read**: partners whose index persons give a self-report of diabetes have a 1.33-fold prevalence of self-reported diabetes than partners whose index persons do not give a self-report of diabetes.

When spousal associations were stratified by duration of marriage, prevalence ratios higher than 1 were observed in all strata in the crude models (Supplementary Table 1a). After confounder adjustment, prevalence ratios are closer to the null effect (supplementary Table 1b). When analysing the effect estimates for married couples only (i.e. 1080 of 1173 couples), effect estimates differed little from the effect estimates for the whole study group (Supplementary Table 2). In separate analyses with only men and only women as index persons, the associations were largely the same for men and women (Supplementary Table 3a and b).

Supplementary Table 4 shows the results of the quantitative bias analysis for the association between self-reported diabetes in the index person and self-reported diabetes in the partner. When non-differential misclassification was assumed, the true prevalence ratios were slightly higher than the observed prevalence ratio, which was 1.33. When differential misclassification was assumed, the true prevalence ratios were lower than the observed prevalence ratio. With an assumed specificity of 0.98 (1.0), an assumed sensitivity of type-2 diabetes of 0.85 when the spouse had type-2 diabetes, and an assumed sensitivity of 0.80 when the spouse did not have type-2 diabetes, the true prevalence ratio was 1.29 (1.19). The true prevalence ratio was 1.13 (1.06) when the corresponding sensitivities were 0.90 and 0.80 (Supplementary Table 4).

## Discussion

Spousal associations examined in the present study were weak to moderate for type-2 diabetes and for the metabolic syndrome in crude models. There were poor associations in adjusted models. The strongest spousal association was found between prediabetes or type-2 diabetes in the index persons and type-2 diabetes in the partners. In addition, there were moderate to strong associations between corresponding risk factors of the couples such as low education, obesity, poor diet, and smoking.

### Causal effect versus association

Diabetes is not a contagious disease. However, a causal effect of diabetes in one person on diabetes in the partner is conceivable if the diagnosis of diabetes in the index person leads the partner to become aware of his or her risk of diabetes and to change his or her lifestyle. Moreover, spousal associations of type-2 diabetes can be explained by confounding, i.e. by shared environment such as air pollution or stress, shared lifestyle such as smoking or criteria in the choice of the partner^20^. Therefore, the observed associations are mainly due to shared lifestyle factors or shared environment. For clinical practice, unadjusted spousal associations are of interest. What a general practitioner or a diabetologist want to know is the extent to which the prevalence of type-2 diabetes is increased in a patient whose partner has diabetes regardless of the factors responsible for the association. As in unadjusted models the prevalence of diabetes is weakly to moderately increased in people whose partners have diabetes, a couple-focused approach may be helpful. Adjusting for shared risk factors may help to disentangle which factors play a role in spousal associations. In our study, age and education played an important role in these associations. Adding lifestyle factors (alcohol, dietary pattern index, smoking, sports) to age and education as potential confounders did not have a strong additional effect on the prevalence ratios. This suggests that lifestyle factors are correlated with age and education, and that a lot of information about lifestyle factors is included in age and education.

### Comparison with earlier studies

Compared with previous meta-analyses by Leong^5^ (Odds Ratio (OR) = 1.26 [95% CI 1.08–1.45]) or Appiah^6^ (OR = 1.88 [95% CI 1.52–2.33]) the results of the present study on spousal associations of type-2 diabetes are less strong. However, in the meta-analysis of Appiah, an I^2^ of 98% indicated considerable heterogeneity, as shown by 7 out 17 studies outside the ± 2 standard deviations parallel lines. When these seven studies (including Cunningham^10^ (OR = 8.7 [95% CI 7.4–10.2]) and Mothojakan^36^ (OR = 7.2 [95% CI 2.9–18.0]) were excluded, the OR was only 1.33 [95% CI 1.24–1.42]. Studies published after the meta-analysis in 2019 also showed heterogeneous results: Hazard Ratios (HRs) were 1.07 (0.75–1.53) for men and 1.34 (1.01–1.79) for women in Iran, an OR = 1.45 (1.34–1.58) was reported for Japan, and an OR = 1.82 (1.04–3.19) for men and OR = 1.90 (1.09–3.31) for women were observed in Korea^7,8,37^.

To date, little is known about the association of prediabetes in married couples. Kim et al. reported an association between married couples only for fasting glucose ≥ 110 mg/dl (OR = 2.25 [95% CI 1.83–2.75])^14^. Another study from the UK reported a higher chance of (pre)diabetes for partners of persons with diabetes (OR = 2.32 [95% CI 1.87–3.98])^15^. Our study is in line with these earlier results in couples, however, associations were less strong (e.g. PR = 1.20 (95% CI: 1.06–1.35) for the association between type-2 diabetes in the index person and (pre)diabetes in the partner. Early detection of prediabetes can be helpful because persons with prediabetes have been shown to benefit from lifestyle intervention, and, therefore, the risk of progressing to diabetes can be reduced in persons with unknown prediabetes^11,12^.

In the present study, estimates for the spousal association of the metabolic syndrome were lower than those from previous studies in Korea (OR = 1.49 [95% CI 1.25–1.77] for men and OR = 1.49 [95% CI 1.25–1.77] for women)^14^ and in Japan (OR = 1.54 [95% CI 0.62–3.80] for men and OR = 1.52 [95% CI 0.61–3.76] for women)^17^. However, in the present study the precision of the corresponding effect estimate was much higher than in the previously mentioned studies (PR = 1.17 [95% CI 1.03–1.32]). Overall, it must also be taken into account that odds ratios are usually higher than relative risks but are often interpreted as risks or prevalences. The prevalences of the diseases considered in this study are moderate to high, so use of relative risks and prevalence ratios is more appropriate^38–41^.

With the exception of self-reported diabetes, stratification by the duration of marriage did not show that effect estimates are larger for couples with longer duration of marriage. These results were also found in other studies^9,42–44^. The reason may be that in all groups most couples have been married to each other for a very long time – in the present study, duration of marriage is lower than five years in only 18 (1.5%) of the married couples. In the study of Sun et al. the couples have been married for a median of over 30 years^44^. Therefore, it would be interesting to examine the short-term effects of marriage in future studies in a population that also includes more couples with a marriage duration of less than 20 years.

In contrast to the findings of an Iranian study there is no difference in our effect estimates comparing the influence of men on women and vice versa. The couples seem to have a symmetrical relationship with one another while in Iran men have a higher impact on women’s diabetic status possibly due to their dominant role in the family^7^. Asian studies show results similar to ours^14,17,37,44^.

A quantitative bias analysis of our data showed that unawareness of diabetes leads to an overestimation of the association between self-reported diabetes in the index persons and their partners if diabetes in one spouse increases the awareness of diabetes in the other. There is no data to show whether one spouse is more likely to be aware of having diabetes when the other spouse has diabetes. However, if this were the case, the observed association would be partly due to misclassification bias.

To address the rapidly increasing number of incident diabetes cases worldwide simple strategies such as the couple-focused approach to diabetes detection and treatment could play an important role in the public health system in the future. Existing diabetes programs could be extended to include the partner and to check for its glycaemic status as well. This allows early stages of diabetes to be detected and treated. This can lead to less aggressive treatment than if they are discovered later, as well as fewer complications such as secondary diseases and therefore lower mortality.

### Strengths and limitations

A strength of the present study is the large sample size of 1173 couples from two studies in the Ruhr metropolitan region in Germany. Moreover, this study does not only focus on spousal association of type-2 diabetes, but also of prediabetes and the metabolic syndrome. Contrary to other studies defining diabetes only by fasting blood glucose^15^, blood glucose levels, self-report^43^ or medication^8^ alone, the long-term blood glucose value HbA1c as well as the self-report of diabetes and the intake of antidiabetic drugs or insulin are used to define type-2 diabetes in the present study. Using this wider definition of diabetes, less people with diabetes were missed. Furthermore, the long-term blood glucose value HbA1c represents the glucose level of the last three months in contrast to blood glucose which varies even within hours.

There are also some limitations. First, due to the small number of persons with type-1 diabetes, it can be assumed that not all persons with type-1 diabetes were identified. An underestimation of the effect estimates for spousal associations of dysglycemia may be a consequence. Second, due to missing data, it was probably not possible to exclude all partners who already had (pre-)diabetes or metabolic syndrome before marriage. However, it can be assumed that this number is low, as the included couples mostly married at a young age at which the prevalence of (pre-)diabetes and metabolic syndrome is very low. Third, due to a lack of data, it was not possible to exclude consanguineous married couples. However, the number of such marriages is extremely low in Germany. Fourth, we cannot exclude that some confounders were either not measured precisely enough, or that there was misclassification in confounders, or that some confounders were missed. In particular, results of the validation of the food frequency questionnaire had been rather poor with low Kappa values. Stress, particulate matter, distance to work, walkability of the neighbourhood, noise and sleep patterns are risk factors for diabetes not taken into account in the present study^45–48^. Fifth, no statement can be made about the trends in couple relationships that lasted for a shorter period or only a few years, since few couples are married for less than 20 years.

## Conclusion

Overall, our results confirm for the first time for a large German population previous findings on the association of dysglycemia in couples. Partners of patients with type-2 diabetes have a weakly to moderately increased prevalence of type-2 diabetes in crude models. In addition, our study is one of the few to show spousal associations for prediabetes and the metabolic syndrome. Because of the importance of prediabetes as a preliminary stage of diabetes, spousal associations of prediabetes should receive greater attention in future studies. A couple-focused treatment approach could help to detect early stage dysglycemia and to prevent the progression to diabetes. General practitioners could play an important role in this field, as they often know patients and their partners well and have taken care for them over a long period of time.

### Supplementary Information

Supplementary Tables.

## Data Availability

Due to data security reasons (i.e. data contain potentially participant identifying information), the HNR Study does not allow sharing data as a public use file. Data requests can be addressed to: recall@uk-essen.de.
