# Supplementary material for: Aggregation of type-2 diabetes, prediabetes, and metabolic syndrome in German couples
Source: Sci Rep. 2024 Feb 5;14:2984. doi: 10.1038/s41598-024-53417-1 (PMC10844497; doi:10.1038/s41598-024-53417-1)
Supplement: Supplementary file 1 — Supplementary Tables. [file 41598_2024_53417_MOESM1_ESM.docx]

**Aggregation of type-2 diabetes, prediabetes, and metabolic syndrome in German couples: the Heinz Nixdorf Recall and the Heinz Nixdorf Multigeneration Study**

Lara Brieger ^1^, Sara Schramm ^1^, Börge Schmidt ^1^, Ulla Roggenbuck ^1^, Raimund Erbel ^1^, Andreas Stang ^1,2^, Bernd Kowall ^1^

^1^ Institute for Medical Informatics, Biometry and Epidemiology, Medical Faculty, University Duisburg-

Essen, Essen, Germany

^2^ School of Public Health, Department of Epidemiology Boston University, 715 Albany Street, Talbot

Building, Boston, MA 02118, USA

**Supplementary table 1a**. *Unadjusted* prevalence ratios (95% confidence interval) for spousal associations of (pre-)diabetes / metabolic syndrome stratified by duration of marriage: Results from log-binomial regression analysis

| **Exposure Index person** | **Outcome**  **Partner** | **Married for <20 years**  **(n=120)** | **Married for**  **≥20 <40 years**  **(n=290)** | **Married for**  **≥40 years**  **(n=681)** |
| --- | --- | --- | --- | --- |
| Diabetes  (self-report) | Diabetes  (self-report) | 1.10  (0.33-3.66) | 1.37  (0.50-3.73) | 1.36  (0.87-2.14) |
| Diabetes (self-report, drug intake, HbA1c) ^a^ | Diabetes (self-report, drug intake, HbA1c) ^a^ | 1.45  (0.55-3.83) | 0.93  (0.38-2.26) | 1.20  (0.80-1.80) |
| Prediabetes ^b^ | (Pre-)Diabetes ^c^ | 1.20  (0.80-1.81) | 1.20  (0.93-1.57) | 1.07  (0.92-1.24) |
| Diabetes (self-report, drug intake, HbA1c) ^a^ | (Pre-)Diabetes ^c^ | 1.42  (1.02-1.98) | 1.23  (0.93-1.63) | 1.14  (0.99-1.32) |
| (Pre-)Diabetes ^c^ | (Pre-)Diabetes ^c^ | 1.32  (0.90-1.93) | 1.24  (0.98-1.58) | 1.10  (0.96-1.27) |
| (Pre-)Diabetes ^c^ | Diabetes (self-report, drug intake, HbA1c) ^a^ | 1.64  (0.56-4.78) | 1.28  (0.68-2.39) | 1.47  (0.99-2.18) |
| Metabolic syndrome ^d^ | Metabolic syndrome ^d^ | 1.36  (0.96-1.91) | 1.15  (0.87-1.52) | 1.11  (0.96-1.28) |

reference group (“no”),

^a^ diabetes is defined by either self-report, intake of antidiabetics or insulin or HbA1c ≥ 6.5%,

^b^ prediabetes is defined by 5.7% ≤ HbA1c < 6.5%,

^c^ prediabetes or diabetes,

^d^ The metabolic syndrome is defined by a modification of the Joint Interim Statement. [21]

**Supplementary table 1b**. *Adjusted* prevalence ratios (95% confidence interval) for spousal associations of (pre-)diabetes / metabolic syndrome stratified by duration of marriage: Results from log-binomial regression analysis

| **Exposure Index person** | **Outcome**  **Partner** | **Married for <20 years**  **(n=120)** | **Married for**  **≥20 <40 years**  **(n=290)** | **Married for**  **≥40 years**  **(n=681)** |
| --- | --- | --- | --- | --- |
| Diabetes  (self-report) | Diabetes  (self-report) | 0.76  (0.18-3.23) ^e^ | 1.63  (0.50-5.33) ^e^ | 1.14  (0.73-1.78) |
| Diabetes (self-report, drug intake, HbA1c) ^a^ | Diabetes (self-report, drug intake, HbA1c) ^a^ | 1.12  (0.35-3.65) ^e^ | 0.92  (0.33-2.55) ^e^ | 1.03  (0.74-1.43) |
| Prediabetes ^b^ | (Pre-)Diabetes ^c^ | 1.02  (0.58-1.81) ^e^ | 1.15  (0.89-1.47) | 1.04  (0.90-1.20) |
| Diabetes (self-report, drug intake, HbA1c) ^a^ | (Pre-)Diabetes ^c^ | 1.31  (0.77-2.25) ^e^ | 1.07  (0.78-1.46) | 1.09  (0.92-1.28) |
| (Pre-)Diabetes ^c^ | (Pre-)Diabetes ^c^ | 1.11  (0.65-1.90) ^e^ | 1.15  (0.91-1.46) | 1.05  (0.92-1.20) |
| (Pre-)Diabetes ^c^ | Diabetes (self-report, drug intake, HbA1c) ^a^ | 1.42  (0.41-4.88) ^e^ | 1.23  (0.58-2.61) ^e^ | 1.22  (0.87-1.72) |
| Metabolic syndrome ^d^ | Metabolic syndrome ^d^ | 1.31  (0.84-2.03) | 1.05  (0.83-1.34) | 1.01  (0.87-1.17) |

reference group (“no”),

^a^ diabetes is defined by either self-report, intake of antidiabetics or insulin or HbA1c ≥ 6.5%,

^b^ prediabetes is defined by 5.7% ≤ HbA1c < 6.5%,

^c^ prediabetes or diabetes,

^d^ The metabolic syndrome is defined by a modification of the Joint Interim Statement. [21]

^e^ log-linear models with a Poisson working likelihood and robust standard errors were used

Adjustments for age, education, alcohol, dietary pattern index, smoking, sports, BMI, lipids (HDL, LDL, cholesterol, triglycerides)

**Supplementary table 2.** Unadjusted prevalence ratios (95% confidence interval) for spousal associations of (pre-)diabetes / metabolic syndrome: Results from log-binomial regression analysis (*only married couples*, N=1080)

| **Exposure Index person** | **Outcome**  **Partner** | **Number of index persons with exposure in column 1** | **Number of partners with outcome in column 2** | **prevalence ratio (95% CI)** |
| --- | --- | --- | --- | --- |
| Diabetes (self-report) | Diabetes  (self-report) | Yes 153  No 927 (ref) | 25 (16.3%)  106 (11.4%) | 1.43 (0.96-2.13)  1 |
| Diabetes (self-report, drug intake, HbA1c) ^a^ | Diabetes (self-report, drug intake, HbA1c) ^a^ | Yes 180  No 850 (ref) | 31 (17.2%)  125 (14.7%) | 1.17 (0.82-1.68)  1 |
| Prediabetes ^b^ | (Pre-)Diabetes ^c^ | Yes 469  No 381 (ref) | 269 (57.4%)  191 (50.1%) | 1.14 (1.01-1.30)  1 |
| Diabetes (self-report, drug intake, HbA1c) ^a^ | (Pre-)Diabetes ^c^ | Yes 180  No 850 (ref) | 115 (63.9%)  460 (54.1%) | 1.18 (1.04-1.34)  1 |
| (Pre-)Diabetes ^c^ | (Pre-)Diabetes ^c^ | Yes 649  No 381 (ref) | 384 (59.2%)  191 (50.1%) | 1.18 (1.05-1.33)  1 |
| (Pre-)Diabetes ^c^ | Diabetes (self-report, drug intake, HbA1c) ^a^ | Yes 649  No 381 (ref) | 111 (17.1%)  45 (11.8%) | 1.45 (1.05-2.00)  1 |
| Metabolic syndrome ^d^ | Metabolic syndrome ^d^ | Yes 553  No 458 (ref) | 295 (53.3%)  214 (46.7%) | 1.14 (1.01-1.29)  1 |

Ref: reference group, MS: metabolic syndrome,

^a^ diabetes is defined by either self-report, intake of antidiabetics or insulin or HbA1c of ≥ 6.5%,

^b^ prediabetes is defined by 5.7% ≤ HbA1c < 6.5%,

^c^ prediabetes or diabetes,

^d^ The metabolic syndrome is defined by a modification of the Joint Interim Statement. [21]

**Supplementary table 3a.** Unadjusted prevalence ratios (95% confidence interval) for spousal associations of (pre-)diabetes / metabolic *with only men as index persons*: Results from log-binomial regression analysis

| **Exposure**  **Man** | **Outcome**  **Woman** | **Number of men with exposure in column 1** | **Number of women with outcome in column 2** | **prevalence ratio (95% CI)** |
| --- | --- | --- | --- | --- |
| Diabetes  (self-report) | Diabetes  (self-report) | Yes 193  No 980 (ref) | 26 (13.5%)  92 (9.4%) | 1.44 (0.96-2.16)  1 |
| Diabetes (self-report, drug intake, HbA1c) ^a^ | Diabetes (self-report, drug intake, HbA1c) ^a^ | Yes 223  No 895 (ref) | 34 (15.2%)  110 (12.3%) | 1.24 (0.87-1.77)  1 |
| Prediabetes ^b^ | (Pre-)Diabetes ^c^ | Yes 462  No 433 (ref) | 273 (59.1%)  228 (52.7%) | 1.12 (1.00-1.26)  1 |
| Diabetes (self-report, drug intake, HbA1c) ^a^ | (Pre-)Diabetes ^c^ | Yes 223  No 895 (ref) | 143 (64.1%)  501 (56.0%) | 1.15 (1.02-1.28)  1 |
| (Pre-)Diabetes ^c^ | (Pre-)Diabetes ^c^ | Yes 685  No 433 (ref) | 416 (60.7%)  228 (52.7%) | 1.15 (1.04-1.28)  1 |
| (Pre-)Diabetes ^c^ | Diabetes (self-report, drug intake, HbA1c) ^a^ | Yes 685  No 433 (ref) | 105 (15.3%)  39 (9.0%) | 1.70 (1.20-2.41)  1 |
| Metabolic syndrome ^d^ | Metabolic syndrome ^d^ | Yes 644  No 452 (ref) | 326 (50.6%)  188 (41.6%) | 1.22 (1.07-1.39)  1 |

Ref: reference group, MS: metabolic syndrome,

^a^ diabetes is defined by either self-report, intake of antidiabetics or insulin or HbA1c ≥ 6.5%,

^b^ prediabetes is defined by 5.7% ≤ HbA1c < 6.5%,

^c^ prediabetes or diabetes,

^d^ The metabolic syndrome is defined by a modification of the Joint Interim Statement. [21]

**Supplementary table 3b.** Unadjusted prevalence ratios (95% confidence interval) for spousal associations of (pre-)diabetes / metabolic *with only women as index persons*: Results from log-binomial regression analysis

| **Exposure**  **Woman** | **Outcome**  **Man** | **Number of women with exposure in column 1** | **Number of men with outcome in column 2** | **prevalence ratio (95% CI)** |
| --- | --- | --- | --- | --- |
| Diabetes  (self-report) | Diabetes  (self-report) | Yes 118  No 1055 (ref) | 26 (22.0%)  167 (15.8%) | 1.39 (0.96-2.01)  1 |
| Diabetes (self-report, drug intake, HbA1c) ^a^ | Diabetes (self-report, drug intake, HbA1c) ^a^ | Yes 144  No 974 (ref) | 34 (23.6%)  189 (19.4%) | 1.22 (0.88-1.68)  1 |
| Prediabetes ^b^ | (Pre-)Diabetes ^c^ | Yes 500  No 474 (ref) | 311 (62.2%)  269 (56.8%) | 1.10 (0.99-1.22)  1 |
| Diabetes (self-report, drug intake, HbA1c) ^a^ | (Pre-)Diabetes ^c^ | Yes 144  No 974 (ref) | 105 (72.9%)  580 (59.5%) | 1.22 (1.09-1.37)  1 |
| (Pre-)Diabetes ^c^ | (Pre-)Diabetes ^c^ | Yes 644  No 474 (ref) | 416 (64.6%)  269 (56.8%) | 1.14 (1.03-1.25)  1 |
| (Pre-)Diabetes ^c^ | Diabetes (self-report, drug intake, HbA1c) ^a^ | Yes 644  No 474 (ref) | 143 (22.2%)  80 (16.9%) | 1.32 (1.03-1.68)  1 |
| Metabolic syndrome ^d^ | Metabolic syndrome ^d^ | Yes 514  No 582 (ref) | 326 (63.4%)  318 (54.6%) | 1.16 (1.05-1.28)  1 |

Ref: reference group, MS: metabolic syndrome,

^a^ diabetes is defined by either self-report, intake of antidiabetics or insulin or HbA1c ≥ 6.5%,

^b^ prediabetes is defined by 5.7% ≤ HbA1c < 6.5%,

^c^ prediabetes or diabetes,

^d^ The metabolic syndrome is defined by a modification of the Joint Interim Statement. [21]

**Supplementary table 4.** Corrected prevalence ratios for the association between self-reported diabetes in the index person and self-reported diabetes in the partner under various assumptions about the sensitivity and specificity of self-reported diabetes

| **Sensitivity of diabetes**  **(index person)** | | **Sensitivity of diabetes**  **(partner)** | | **Specificity of diabetes^a^** | **Type of misclassification** | **Prevalence ratio** |
| --- | --- | --- | --- | --- | --- | --- |
| **Diabetes in partner** | | **Diabetes in index person** | |  |  |  |
| **yes** | **no** | **yes** | **no** |  |  |  |
| 1 | 1 | 1 | 1 | 1 | - | 1.33^b^ |
| 0.9 | 0.9 | 0.9 | 0.9 | 1 | nd | 1.34^c^ |
| 0.8 | 0.8 | 0.8 | 0.8 | 1 | nd | 1.35 |
| 0.9 | 0.8 | 0.9 | 0.8 | 1 | d | 1.06 |
| 0.85 | 0.8 | 0.85 | 0.8 | 1 | d | 1.19 |
| 0.9 | 0.9 | 0.9 | 0.9 | 0.98 | nd | 1.47 |
| 0.8 | 0.8 | 0.8 | 0.8 | 0.98 | nd | 1.48 |
| 0.9 | 0.8 | 0.9 | 0.8 | 0.98 | d | 1.13 |
| 0.85 | 0.8 | 0.85 | 0.8 | 0.98 | d | 1.29 |

nd: non-differential misclassification; d: differential misclassification

^a^ for diabetes in the index person and diabetes in the partner

^b^ observed prevalence ratio

^c^ **How to read**: If the sensitivity of self-reported diabetes were 0.9 in the index person (regardless of whether the partner had diabetes or not), and if the sensitivity of self-reported diabetes were 0.9 in the partner (regardless of whether the index person had diabetes or not), and the specificity of self-reported diabetes were 1, the true prevalence ratio would be 1.34.
